# Extraction, Characterization and Immunological Activity of Polysaccharides from *Rhizoma gastrodiae*

**DOI:** 10.3390/ijms17071011

**Published:** 2016-06-25

**Authors:** Juncheng Chen, Shan Tian, Xiaoying Shu, Hongtao Du, Na Li, Junru Wang

**Affiliations:** 1 Shaanxi Key Laboratory of Natural Products & Chemical Biology, College of Sciences, Northwest A & F University, Yangling 712100, Shaanxi, China; cjc2009@nwsuaf.edu.cn (J.C.); stian@nwsuaf.edu.cn (S.T.); shuxiaoying1@163.com (X.S.); duht@nwsuaf.edu.cn (H.D.); lnuk@nwsuaf.edu.cn (N.L.); 2 College of Plant Protection, Northwest A & F University, Yangling 712100, Shaanxi, China

**Keywords:** *Rhizoma gastrodiae*, polysaccharides, optimized extraction, characterization, immunological activity

## Abstract

A response surface and Box-Behnken design approach was applied to augment polysaccharide extraction from the residue of *Rhizoma gastrodiae*. Statistical analysis revealed that the linear and quadratic terms for three variables during extraction exhibited obvious effects on extraction yield. The optimum conditions were determined to be a liquid-to-solid ratio of 54 mL/g, an extraction temperature of 74 °C, an extraction time of 66 min, and three extractions. These conditions resulted in a maximum *Rhizoma gastrodiae* polysaccharide (RGP) extraction yield of 6.11% ± 0.13%. Two homogeneous polysaccharides (RGP-1a and RGP-1b) were obtained using DEAE cellulose-52 and Sephadex G-100 columns. The preliminary characterization of RGP-1a and RGP-1b was performed using HPLC-RID, HPGPC, and FTIR. Tests of the immunological activity *in vitro* showed that the two polysaccharides could significantly stimulate macrophages to release NO and enhance phagocytosis in a dose-dependent manner. In particular, RGP-1b (200 μg/mL) and LPS (2 μg/mL) had almost the same influence on the NO production and phagocytic activity of RAW 264.7 macrophages (*p* > 0.05). All the data obtained indicate that RGP-1a and RGP-1b have the potential to be developed as a health food.

## 1. Introduction

Polysaccharides constitute long chains or molecular strands of monosaccharides connected by glycosidic linkages. These polymeric carbohydrate arrangements are found almost exclusively in animals, plants, and microbes. Polysaccharides from plants have diverse bioactivity and high safety in human beings, and the isolation of polysaccharides from herbal or agricultural material has become an important area of research. A number of studies have shown that polysaccharides have various medicinal properties, including immunological [1,2,3], anti-cancer [4,5,6], anti-oxidant [7,8,9], anti-inflammatory [10,11] and hypoglycemic activity [12,13].

*Rhizoma gastrodiae* (called *Tianma* in Chinese) is the dried rhizome of *Gastrodiaelata* Blume (Orchidaceae). In China, it is used as both a traditional medicine and a food because of its diverse biological activity. For centuries, it has been used to treat convulsion, neurasthenia, headache, ischemia, vertigo, dementia, and hemiplegia [14,15,16,17]. Phenolic compounds, such as gastrodin, 4-hydroxybenzyl alcohol and parishin B, are the main bioactive constituents of rhizoma gastrodiae [18,19,20], and recent research has indicated that *Rhizoma gastrodiae* polysaccharide (RGP) is also an important active substance. Ming’s team [21] isolated the polysaccharide PGEB-3H from *Rhizoma gastrodiae* and found that it has hypolipidemic activity, while Chen’s group [22] reported that a RGP has anti-cancer activity. After phenolic compounds are extracted from *Rhizoma gastrodiae*, the raw residue is discarded, which might be a useful polysaccharides resource as it contains plenty of RGP. Although some studies have been done on RGP, reports on the structure and activity of this polysaccharide are limited.

Therefore, to enhance RGP resource efficiency, we applied response surface methodology (RSM) to improve polysaccharide extraction from *Rhizoma gastrodiae* residue, and to provide more evidence to support the use of RGP in the food industry. In addition, basic structural characterizations of RGP-1a and RGP-1b were under taken using a combination of chemical and instrumental analyses, and the immunological activity of RGP-1a and RGP-1b was examined further.

## 2. Results and Discussion

### 2.1. Single-Factor Experimental Analysis

#### 2.1.1. Effect of Extraction Time on the Yield of *Rhizoma gastrodiae* Polysaccharide (RGP)

**Figure 1:**
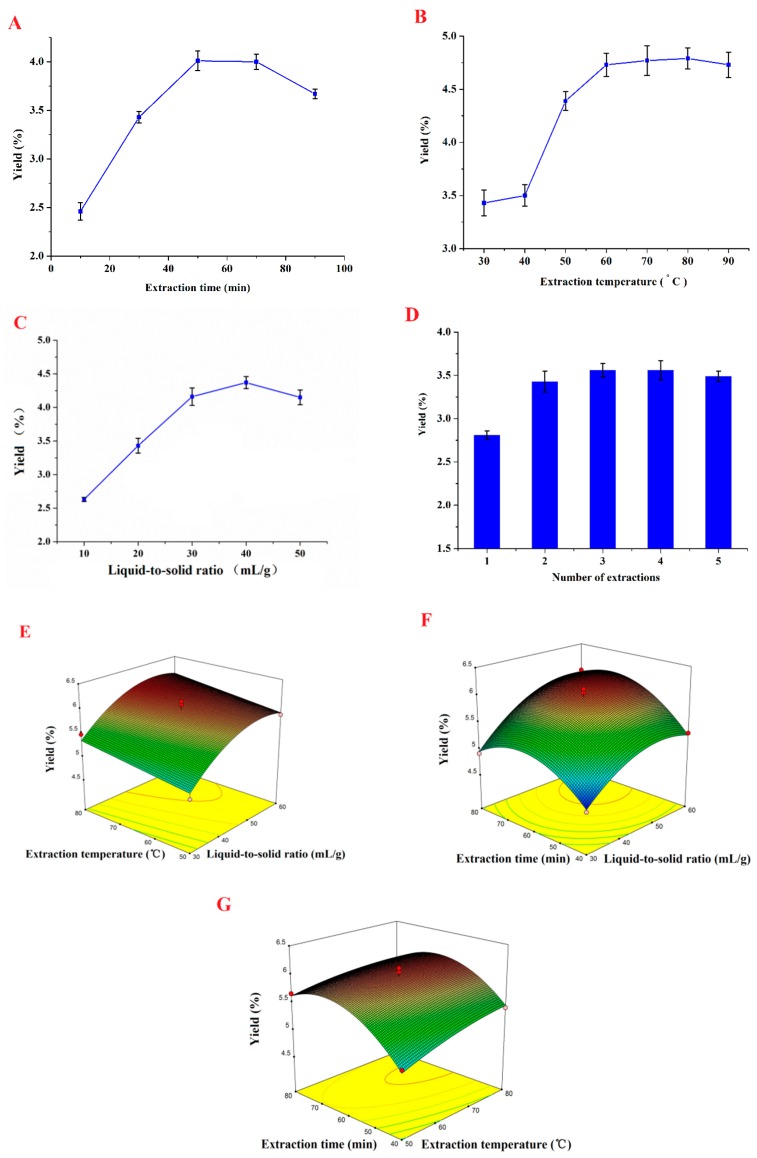
Effects of extraction time (**A**); extraction temperature (**B**); liquid-to-solid ratio; (**C**) and number of extractions (**D**) on the extraction yield of RGP. Each value is presented as mean ± S.D. (*n*= 3); Response surface (3D) showing the effects of extraction temperature and liquid-to-solid ratio at a fixed extraction time of 60 min (**E**); extraction time and liquid-to-solid ratio at a fixed extraction temperature 65 °C (**F**); extraction time and extraction temperature at a fixed liquid-to-solid ratio of 45 mL/g on extraction yield of RGP (**G**).

The effect of different extraction times (10, 30, 50, 70 and 90 min) on the extraction yield of RGP was determined, with the other extraction parameters fixed as follows: the liquid-to-solid ratio was 30 mL/g, the extraction temperature was 60 °C, and the number of extractions was two. As shown in Figure 1A, the yield significantly increased when the extraction time was increased from 10 to 50 min. However, the yield decreased when the extraction time was above 70min, because too long an extraction time can lead to degradation of polysaccharide [23]. So, the favored extraction time is 40–70 min.

#### 2.1.2. Effect of Extraction Temperature on the Yield of RGP

We determined the RGP extraction yield under a series of different extraction temperatures (30, 40, 50, 60, 70, 80 and 90 °C), but fixed liquid-to-solid ratio (30 mL/g) and water extraction time (60 min), with extractions performed in duplicate. As shown in Figure 1B, the yield gradually increased as the extraction temperature rose from 30 to 80 °C, after which there was no significant change. The extraction coefficient is enhanced by a higher extraction temperature due to an increase in polysaccharide solubility [24]. An extraction temperature range of 50–80 °C was thus considered to be optimum.

#### 2.1.3. Effect of the Liquid-to-Solid Ratio on the Yield of RGP

The liquid-to-solid ratio significantly affects the extraction yield of RGP. A low ratio can result in incomplete polysaccharide extraction, whereas a high ratio can lead to increased process costs. We determined the RGP extraction yield under a series of liquid-to-solid ratios (10, 20, 30, 40, 50 and 60 mL/g), but fixed extraction time (60 min) and temperature (60 °C), with extractions performed in duplicate. As shown in Figure 1C, extraction yield increased considerably with the increase in the liquid-to-solid ratio (10–40 mL/g), but no obvious change was observed when the ratio was further increased. Based on these results, a range of 30–60 mL/g was applied in our work.

#### 2.1.4. Effect of the Number of Extractions on the Yield of RGP

The efficiency of different numbers of extractions (1–5) on the extraction yield of RGP was investigated, with the other extraction conditions fixed as follows: extraction temperature 60 °C, extraction time 30 min, and liquid-to-solid ratio 30 mL/g. The results (Figure 1D) show that the extraction yield of RGP increases as the number of extractions is increased from one to three, but there is no further increase with additional extractions. Therefore, the optimum number of extractions is three.

#### 2.1.5. Optimum Conditions for the RSM Experiments

The single-factor experimental analysis indicated that the optimum conditions for the RSM experiments are an extraction time of 40–70 min, an extraction temperature of 50–80 °C, a liquid-to-solid ratio of 30–60 mL/g and three extractions.

### 2.2. Optimization of the RGP Extraction

#### 2.2.1. Predicted Model and Statistical Analysis

The Box-Behnken design matrix and the results of the RSM experiments carried out to determine the effects of the three independent variables are given in Table 1. By applying multiple regression analysis to the experimental data, the response variable (*Y*) can be characterized by the following second-order polynomial equation:

(1) $$Y=5.95+0.39X_{1}+0.15X_{2}+0.25X_{3}-0.08X_{1}X_{2}+0.095X_{1}X_{3}-0.075X_{2}X_{3}-0.33X_{1}^{2}-0.075X_{2}^{2}-0.43X_{3}^{2}$$

where *Y* is the extraction yield (%) of RGP, and *X*_1_, *X*_2_ and *X*_3_ are the coded variables for the liquid-to-solid ratio, extraction temperature, and extraction time, respectively.

We used analysis of variance (ANOVA) to determine the appropriateness of the proposed model and identify the significant factors from the four. The results showed that the predicted model can be summarized as in Table 2. The *R*^2^ and *R*^2^_adj_ values for the model (0.9784 and 0.9507, respectively) did not differ greatly, which indicated close agreement between the experimental results and the theoretical values obtained using the polynomial model. A low *p* value (*p* < 0.0001) confirmed that the model could represent the actual relationship between the parameters and the response with significance. Furthermore, the model exhibited a high degree of accuracy, consistency, and reproducibility, as evidenced by the calculated coefficient of variation (1.86%).

The *p* values were used as a tool to evaluate the significance of each coefficient. The smaller the *p* value, the more significant the corresponding coefficient [25]. As can be seen in Table 2, the coefficients for the linear terms (*X*_1_, *X*_2_ and *X*_3_) and the quadratic terms ($X_{1}^{2}$ and $X_{3}^{2}$) were highly significant (*p* < 0.01). The coefficients for the other terms were not significant (*p* > 0.05).

#### 2.2.2. Analysis of the Response Surface

The three-dimensional (3D) response surfaces were plotted using Design-Expert software (version 8.0.6, Statease, MN, USA) in order to study the effects of the parameters (liquid-to-solid ratio, extraction temperature, and extraction time) and their interactions on the extraction yield of RGP. The results are presented in Figure 1E–G.

Figure 1E shows the effect of the liquid-to-solid ratio and the extraction temperature on the extraction yield of RGP at a fixed extraction time of 60 min. It can be seen that the maximum yield can be achieved when the liquid-to-solid ratio is 53.4 mL/g and the extraction temperature is 73.5 °C.

Figure 1F shows the 3D response surface plot of the extraction yield of RGP when varying the liquid-to-solid ratio and the extraction time but keeping the extraction temperature fixed at 65 °C. It can be observed that maximum yield was obtained at a liquid-to-solid ratio of 53.4 mL/g and an extraction time of 66.1 min.

Figure 1G shows the effects of extraction temperature and time on RGP yield at a liquid-to-solid ratio of 45 mL/g, under which maximum yield was reached at an extraction temperature of 73.5 °C and extraction time of 66.1 min.

#### 2.2.3. Verification of the Model

To verify and validate the model equation, we performed five experiments to assess the optimal variables for extraction under a liquid-to-solid ratio of 54 mL/g, extraction time of 66 min, and extraction temperature of 74 °C. The extractions were carried out in triplicate. There was no significant difference (*p* > 0.05) between the experimentally determined mean yield (6.11% ± 0.13%) and the predicted yield (6.15%).Therefore, the results suggest that the model is accurate and adequate for modeling the extraction of RGP.

### 2.3. Purification and Preliminary Characterization of RGP

#### 2.3.1. Purification, Homogeneity, and Molecular Weights of RGP-1a and RGP-1b

**Figure 2:**
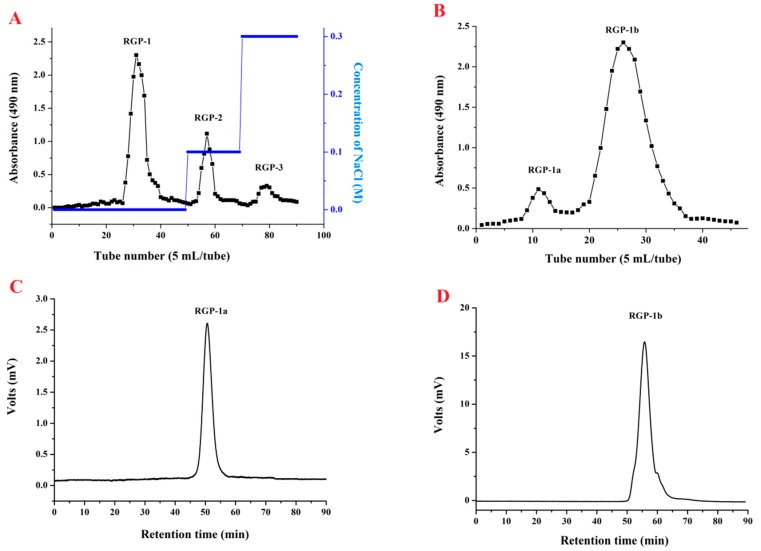
(**A**) Elution curve of crude RGP on DEAE-cellulose column; (**B**) elution curve of RGP-1 on Sephadex G-100 column; (**C**) HPGPC spectra of RGP-1a and (**D**) RGP-1b.

The crude RGP was prepared using the optimal extraction conditions. It was then dissolved in distilled water and fractionated into three fractions (RGP-1, RGP-2, and RGP-3) using a DEAE cellulose-52 column (Figure 2A). The RGP-1 fraction was further separated using a Sephadex G-100 column, resulting in two fractions (RGP-1a and RGP-1b) (Figure 2B).

High-performance gel permeation chromatography (HPGPC) was used to determine the homogeneity and the molecular weights of RGP-1a and RGP-1b. As shown in Figure 2C,D, both RGP-1a and RGP-1b demonstrated a single and symmetrical peak, indicating that they were homogeneous polymers. Using the calibration curve, the average molecular weights of RGP-1a and RGP-1b were calculated to be 19.25 and 3.92 kDa, respectively. These molecular weights are lower than those of polysaccharides extracted from *Rhizoma gastrodiae* reported in the literature, 700 [22] and 28.8 kDa [21], suggesting that RGP-1a and RGP-1b may be two novel polysaccharides from rhizome gastrodiae.

#### 2.3.2. Preliminary Characterization of RGP-1a and RGP-1b

**Figure 3:**
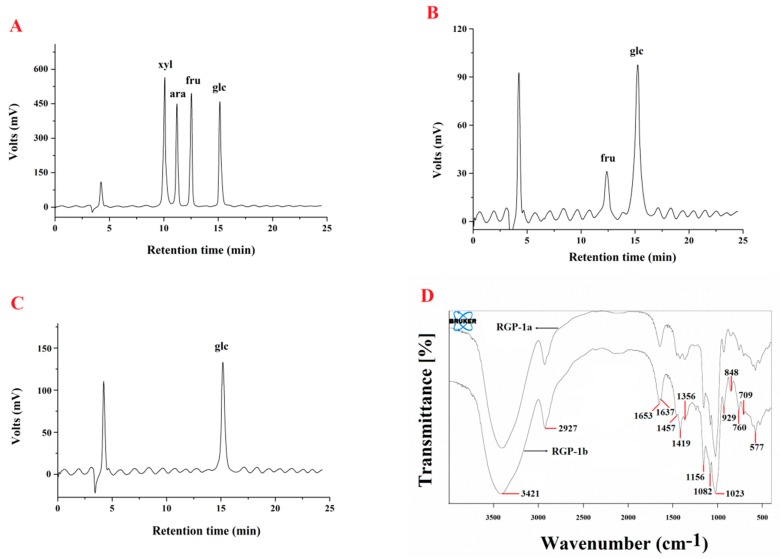
(**A**) HPLC spectra of standard monosaccharide; (**B**) RGP-1a; (**C**) RGP-1b and (**D**) FTIR spectra of RGP.

The carbohydrate content of RGP-1a and RGP-1b was 92.53% and 96.88%, respectively, and the two polysaccharides were free of protein and uronic acid. The monosaccharide compositions of RGP-1a and RGP-1b were analyzed using high-performance liquid chromatography-refractive index detector (HPLC-RID)(Figure 3A–C).The results showed that RGP-1a was composed of fructose and glucose in a molar ratio of 1:10.68, and RGP-1b consisted mainly of glucose. To the authors’ knowledge, it is the first time fructose is identified in this polymer.

Fourier transform infrared (FTIR) spectra of RGP-1a and RGP-1b were recorded over the range of 400–4000 cm^−1^ (Figure 3D). The strong absorption peak around 3421 cm^−1^ and the band around 2927 cm^−1^ were attributed to the –OH and C–H stretching vibrations, respectively. The absorption peak at around 1653 cm^−1^ is due to associated water [21,26]. The band in the region 1365–1457 cm^−1^ was attributed to the characteristic of –OH vibrations and C–H bending vibrations. The absorption peaks at 1023, 1082 and 1156 cm^−1^ suggest the presence of C–O–C and C–O–H linkages, indicating a pyranose form of the glucosyl residue. Absorption at 929.0 cm^−1^ is typical of d-glucose in pyranose form. Moreover, the characteristic absorption bands at 848 cm^−1^ imply that the two purified fractions contain *α*-glycosidic bonds [21].

### 2.4. Effects of RGP-1a and RGP-1b on Macrophages in Vitro

#### 2.4.1. Effects of RGP-1a and RGP-1b on Macrophage Viability

**Figure 4:**
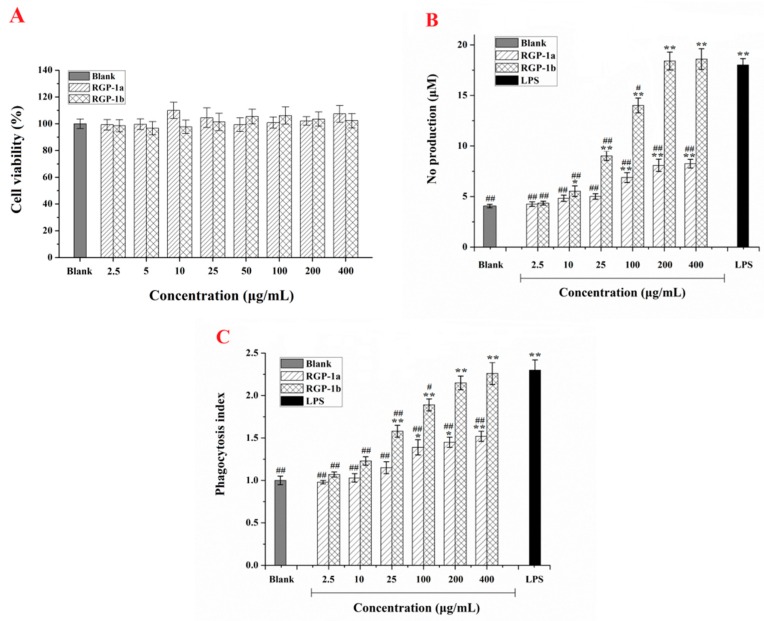
Effects of RGP-1a and RGP-1b on proliferation (**A**); NO production (**B**); and phagocytosis index (**C**) of RAW 264.7 cells. Cells were treated with RGP-1a and RGP-1b at various concentrations for 24 h. The values are presented as mean ± S.D. (*n* = 5). * *p* < 0.05, ** *p* < 0.01 compared with blank group; ^#^ *p* < 0.05, ^##^ *p* < 0.01 compared with LPS (2 μg/mL) group.

Macrophages are a critical part of the natural immune defense system of hosts, which has various immune regulatory functions [27]. Therefore, it is important to know the cytotoxic effects of RGP-1a and RGP-1b on RAW 264.7 cells. As shown in Figure 4A, compared with the blank control, neither RGP-1a nor RGP-1b significantly affected the viability of RAW 264.7 cells over the whole concentration range of RGP analyzed (2.5–400 μg/mL). According to the research conducted by Zhang’s team [28], this indicates that the two polysaccharides from RGP are not toxic to RAW 264.7 cells. Thus, both NO production and phagocytosis satisfactorily represented cell function without influencing cell quantity [28].

#### 2.4.2. Effects of RGP-1a and RGP-1b on Macrophage Activation

The application of macrophage activation is a novel approach in immunotherapy disease treatment. Once macrophages are activated they can release a significant amount of NO, which promotes tumor cell and pathogen death, and then acts as an intracellular messenger molecule to regulate numerous biological functions [29]. RAW 264.7 cells were treated with RGP-1a and RGP-1b at various concentrations and cultivted for 24 h. As shown in Figure 4B, both RGP-1a and RGP-1b stimulated NO production in the cells in a dose-dependent manner. Compared with the blank control, the production of NO increased significantly (*p* < 0.05) with increasing concentrations of the two polysaccharides (100–400 μg/mL). Nevertheless, the effect of low concentrations of RGP-1a (2.5–25 μg/mL) was not significant (*p* > 0.05). Moreover, the production of NO when cells were treated with 200 μg/mL RGP-1b was equivalent to that seen with the positive control (lipopolysaccharide (LPS), 2 μg/mL), which was better than the polysaccharides (ACPS-1 and ACPS-2) isolated from the roots of Actinidia Chinensis with 300 μg/mL [28]. The results confirm that the two polysaccharides activate macrophages and increase the production of NO in RAW 264.7 cells in a dose-dependent manner. This may be one of the reasons why RGP has antitumor activity.

#### 2.4.3. Effects of RGP-1a and RGP-1b Macrophages Phagocytosis

As a defense response, macrophages can phagocytose (that is, envelop and destroy) foreign material as well as aging bacteria, damaged cells, and necrotic tissues, with phagocytic ability considered to be a strong indicator of macrophage activation [29]. In the present study, phagocytic activity of RAW 264.7 macrophages under RGP-1a and RGP-1b treatment was evaluated using the uptake of neutral red. The phagocytosis index (PI) of the two polysaccharides increased in a dose-dependent manner over the concentration range 2.5–400 μg/mL, the effect of RGP-1b being better than that of RGP-1a (Figure 4C). Nevertheless, compared with the blank control, the PIs of macrophages treated with lower concentrations of the polysaccharides were not significantly affected (*p* < 0.05). Furthermore, there was no significant difference (*p* > 0.05) between the PI for RGP-1b at a concentration of 200 μg/mL and the PI for the positive control (LPS, 10 μg/mL). The results aresimilar to the effect of LP1 on the phagocytic ability of macrophages [30], which indicate that, within a certain concentration range, RGP-1a and RGP-1b significantly enhanced RAW 264.7 macrophage phagocytosis. This finding might relate to the two polysaccharides being bound by a particular receptor on the macrophage surface [31,32].

## 3. Materials and Methods

### 3.1. Materials

*Rhizoma gastrodiae* was purchased from the Shaanxi Han Wang Pharmaceutical Co. (Shaanxi, China). The samples were ground and passed through an 80-mesh sieve, and the *Rhizoma gastrodiae* powder preserved at 4 °C. Lipopolysaccharide (LPS) sodium nitrite, 3-(4,5-dimethyltiazol-2-yl)-2,5 diphenyltetrazolium bromide (MTT) and Griess reagent (1% sulfanilamide, 0.1% *N*-(1-Naphthyl) ethylenediamine dihydrochloride and 2.5% phosphoric acid) were purchased from Sigma Chemical Co. (St. Louis, MO, USA). Neutral red and standard monosaccharides were obtained from Aladdin Chemical Reagent Co., Ltd. (Shanghai, China). RAW 264.7 cell lines were provided by the Polysaccharide Biological Functional Laboratory (Northwest A&F University, Shaanxi, China). Trifluoroacetic acid (TFA, Sigma), dimethyl sulfoxide (DMSO, Sigma), ethyl alcohol (Sigma) and other reagents were all of analytical grade. High purity water was prepared in the authors′ laboratory.

### 3.2. Extraction and Determination of Polysaccharide

*Rhizoma gastrodiae* powder was extracted three times with 75% ethyl alcohol (% *v*/*v*) over 24 h. Subsequently, the residue was separated from the ethyl alcohol solvent by centrifugation (3500× *g* for 10 min) and dried at 60 °C for 24 h. The *Rhizoma gastrodiae* residue was extracted using water, and the optimized extraction time and temperature, liquid-to-solid ratio, and number of extractions. The suspension was then centrifuged at 3500× *g* for 20 min. The aqueous extraction solution was disposed using the Sevage method [33] to remove protein, and then concentrated in a rotary evaporator at 60 °C under reduced pressure. The aqueous extraction solution protein removed was precipitated by the addition of absolute ethyl alcohol to a final concentration of 80% (% *v*/*v*) ethanol, and centrifuged again to collect the precipitate. RGP was obtained after freeze drying at −40 °C under vacuum.

We determined the polysaccharide sugar content using phenol-sulfuric acid with d-glucose as the standard [34]. The percentage total polysaccharide yield was calculated as follows:

(2) $$Yield(\%)=\frac{cW_{2}}{W_{1}}\times 100\%$$

where *W*_1_ is the weight of the *Rhizoma gastrodiae* residue obtained after extracted three times with 75% ethyl alcohol (g), *W*_2_ is the weight of crude RGP (g), and *c* is the sugar content in the crude RGP (%).

### 3.3. Experimental Design

Based on the results of the single-factor experiments, the RSM coupled with a three-factor, three-level Box–Behnken design was employed in the optimization procedure [35]. The liquid-to-solid ratio (*X*_1_), extraction temperature (*X*_2_), and extraction time (*X*_3_) were the independent variables, and the polysaccharide yield (*Y*) was the response variable of the design experiments. The coded levels of the independent variables are displayed in Table 1. The experiment was carried out at random in order to minimize the effect of unexplained variability. The variables were coded according to the following equation:

(3) $$x_{i}=\frac{X_{i}-X_{0}}{\Delta X}$$

where *x_i_* is the (dimensionless) coded value of the variable *X_i_*, *X*_0_ is the value of *X_i_* at the center point, and ∆X is the step change.

Experimental data from the Box–Behnken design were fitted to an empirical second-order polynomial model, as follows:

(4) $$Y=A_{0}+\sum_{i=1}^{3}A_{i}X_{i}+\sum_{i=1}^{3}A_{ii}X_{i}^{2}+\sum_{i=1}^{2}\sum_{j=i+1}^{3}A_{ij}X_{i}X_{j}$$

where *Y* is the dependent variable (yield of polysaccharide as a real value), *A*_0_ is a constant, *A_i_*, *A_ii_*, and *A_ij_* are coefficients estimated using the model, and *X*_i_ and *X*_j_ are levels of the independent variables.

The effect of each independent variable on a response was evaluated using the model. Design-Expert software (version 8.0.6, Stat-Ease Inc., Minneapolis, MN, USA) was used to design the experiment, calculate the predicted data, and estimate the response of the independent variables. Three additional experiments were done under optimal conditions to verify the validity of the model.

### 3.4. Purification of RGP

After the production of RGP under optimal conditions, the crude RGP was dissolved in deionized water (50 mg/mL) and applied to a DEAE cellulose-52 column (2.6 cm × 80 cm), which was stepwise eluted with 0, 0.1 and 0.3 M sodium chloride (NaCl) solution at a flow rate of 1 mL/min. The eluate (5 mL/tube) was collected automatically, and the carbohydrate content determined using the phenol–sulfuric acid method. Three completely separated fractions (RGP-1, RGP-2 and RGP-3) were obtained. RGP-1 was applied to a Sephadex G-100 column (1.8 cm × 100 cm), which was eluted with deionized water at a 0.2 mL/min flow rate. The elution was checked as described above. The two purified fractions were collected, concentrated, and lyophilized, thus producing two purified polysaccharides (RGP-1a and RGP-1b) for further study.

### 3.5. Characterization of RGP

#### 3.5.1. Carbohydrate, Protein and Uronic Acid Determination

The carbohydrate content of RGP-1a and RGP-1b were determined using the phenol–sulfuric acid method [34] with d-glucose as the standard. Bradford′s method was used to measure the protein content [36], with bovine serum albumin as the standard. The uronic acid content was evaluated using the method described by Blumen Krantz and Asboe-Hansen [37], with galacturonic acid as the standard.

#### 3.5.2. Homogeneity and Molecular Weight Determination

We analyzed the homogeneity and molecular weight of RGP-1a and RGP-1b using high-performance gel permeation chromatography (HPGPC). The Waters 600 HPLC System (Waters corporation, Milford, MA, USA) equipped with a 2414 differential refractive index detector (RID) and three columns in series (Waters Ultrahydrogel 250, 1000 and 2000; 30 cm × 7.8 mm; 6 μm particles) [34]. The columns were calibrated using T-series dextrans (molecular weights 5.2, 10, 48.6, 668 and 2000 kDa) and eluted with sodium acetate (3 mM) at a flow rate of 0.5 mL/min. The log (molecular weight) versus elution time (*t*) calibration curve was based on the following equation:

log(*M*_W_) = − 0.136*t* + 10.94 (5)

#### 3.5.3. Analysis of Monosaccharide Composition

The RGP-1a and RGP-1b monosaccharide compositions were evaluated using HPLC-RID (LC-15C, Shimadzu, Japan). Briefly, the 200 μL polysaccharide solution (5 mg/mL) was hydrolyzed with 200 μL of 4 Mtrifluoroacetic acid (TFA) at 120 °C for 6 h. After hydrolysis, the solution was evaporated under vacuum. To remove TFA, the residue was dissolved in methanol (3 mL) and evaporated to dryness. Subsequently, the dried sample was dissolved with 75% acetonitrile (aq.). Finally, the solution was filtered through a 0.45 μm filter and analyzed using HPLC. A Shim-pack CLC–NH_2_ column was used, and the chromatographic conditions were as follows: column temperature 30 °C, injection volume 20 μL, mobile phase 75% acetonitrile (aq.), and a RID-10A refractive index detector (Shimadzu, Japan).

#### 3.5.4. FTIR Spectrometric Analysis

We mixed the dried polysaccharides with KBr powder (spectroscopic grade), then ground and pressed the mixture into pellets (1 mm) for further FTIR measurement. The IR spectra of RGP-1a and RGP-1b were recorded using a FTIR spectrophotometer (Bruker Tensor 27, Bruker, Germany) the frequency range 400–4000 cm^−1^.

### 3.6. Effect of Polysaccharides on Macrophage Cell

#### 3.6.1. RAW 264.7 Cell Viability Assays

An MTT-based colorimetric method was used to detect the effects of RGP-1a and RGP-1b on RAW 264.7 cell viability [38]. The macrophage cells were placed in 96-well plates (5 × 10^5^ cells/well) and incubated for 12 h at 37 °C in a 5% CO_2_ humidified incubator. The medium was then discarded and different concentrations of RGP-1a or RGP-1b (2.5, 5, 10, 25, 50, 100, 200 and 400 μg/mL) were added to the RAW 264.7 cells. The cells were then incubated for a further 24 h. The medium was used as the blank control. After incubation, MTT solution (20 μL/well) was added to each well, and the cells then incubated for a further 4 h. The supernatant was discarded, 150 μL of DMSO was added, and the cells incubated for another 10 min. The absorbance was measured at 570 nm using a microplate reader (Perlong DNM-9062, Beijing, China).

#### 3.6.2. Nitric Oxide (NO) Production Assay

Griess reagent was employed to measure NO production [39]. The RAW 264.7 cells (1 × 10^6^ cells/well) were incubated in a 5% CO_2_ humidified incubator for 12 h at 37 °C. After that, the cells were treated with various concentrations of RGP-1a or RGP-1b (2.5, 10, 25, 100, 200 and 400 μg/mL), and then incubated for a further 24 h. The medium and LPS (2 μg/mL) were used as the blank and positive controls, respectively. After incubation, 100 μL of culture supernatant was mixed with an equal volume of Griess reagent in 96-well plates before being incubated in a 5% CO_2_ humidified incubator for 10 min at 25 °C. The absorbance was measured at 540 nm using a microplate reader. The concentration of nitrite in the culture supernatants was measured to assess the NO production of the RAW 264.7 cells, and the concentrations of NO^2−^ were determined by least-squares linear regression analysis of the sodium nitrite standard curve.

#### 3.6.3. Assay of Macrophages Phagocytosis

The phagocytic ability of RAW 264.7 cells was determined following the uptake of neutral red [37]. Briefly, the cells (1 × 10^6^ cells/well) were dispensed into 96-well plates and incubated for 12 h. The cells were then treated with various concentrations of RGP-1a or RGP-1b (2.5, 10, 25, 100, 200 and 400 μg/mL), and incubated for a further 24 h. The medium and LPS (2 μg/mL) were used as the blank and positive control, respectively. Subsequently, 0.075% neutral red solution (100 μL/well) was added to the cells, which were then incubated for 1 h. After wards, the medium was removed and the cells were washed three times with PBS. Cell lysis buffer (1% glacial acetic acid: ethanol = 1:1, 100 μL/well) was subsequently added for cellysation. After 15 h incubation at room temperature, the optical density of the cells was measured at 540 nm using a microplate reader. The phagocytosis index (PI) was determined as per the following equation:

(6) $$\text{PI}\;=\frac{A_{1}}{A_{0}}$$

where *A*_0_ is the absorbance of the blank control and *A*_1_ is the absorbance of sample.

### 3.7. Statistical Analysis

All data are presented as means ± standard deviations. Significant differences between results were examined by ANOVA using SPSS 13.0 (SPSS Inc, Chicago, IL, USA) for Windows. Statistical significance was defined as *p* < 0.05 for all tests.

## 4. Conclusions

In the present study, the process for extracting RGP was successfully optimized through a combination of single-factor experiments and RSM. When the extraction was carried out under the optimum process conditions (extraction temperature 74 °C, extraction time 66 min, liquid-to-solid ratio 54 mL/g, three extractions), a yield of 6.11% was obtained, which is close to the theoretical yield of 6.15%. Two novel homogeneous polysaccharides (RGP-1a and RGP-1b) with average molecular weights of 19.25 and 3.92 kDa, respectively, were purified using DEAE cellulose-52 and Sephadex G-100 columns. The sugar contents of RGP-1a and RGP-1b were 92.53% and 96.88%, respectively, and no uronic acid or protein was found in the two polysaccharides. RGP-1a is composed of fructose and glucose in a molar ratio of 1:10.68; RGP-1b consists mainly of glucose. In addition, it was found that RGP-1a and RGP-1b have a significant effect on the NO production and phagocytic activity of RAW 264.7 macrophages. RGP-1b showed a stronger effect than RGP-1a, which might be due to the different monosaccharide compositions and structures of the two polysaccharides. However, more research into the structure and mechanism of the biological activities of RGP-1a and RGP-1b is required. Nevertheless, the results of the present study indicated that the use of those two polysaccharides isolated from RGP in health foods has promising prospects.

## Figures and Tables

**Table 1:** The BBD matrix and response values for the extraction yield of *Rhizoma gastrodiae* Polysaccharide (RGP).

Run	*X*_1_ (Liquid-to-Solid Ratio, mL/g)	*X*_2_ (Extraction Temperature, °C)	*X*_3_ (Extraction Time, min)	Yield of RGP (%)
1	1 (60)	−1 (50)	0 (60)	5.78
2	−1 (30)	0 (65)	1 (80)	4.91
3	0 (45)	0 (65)	0 (60)	5.84
4	−1 (30)	0 (65)	−1 (40)	4.59
5	0 (45)	0 (65)	0 (60)	6.03
6	1 (60)	0 (65)	1 (80)	5.97
7	−1 (30)	1 (80)	0 (60)	5.47
8	0 (45)	0 (65)	0 (60)	5.91
9	0 (45)	0 (65)	0 (60)	6.10
10	1 (60)	1 (80)	0 (60)	6.01
11	0 (45)	−1 (50)	−1 (40)	5.01
12	0 (45)	1 (80)	1 (80)	5.73
13	0 (45)	0 (65)	0 (60)	5.89
14	0 (45)	−1 (50)	1 (80)	5.66
15	0 (45)	1 (80)	−1 (40)	5.38
16	1 (60)	0 (65)	−1 (40)	5.27
17	−1 (30)	−1 (50)	0 (60)	4.92

**Table 2:** Analysis of variance (ANOVA) results of the regression model for the extraction yield of RGP.

Source	Sum of Squares	Df	Mean Square	*F*-Value	*p*-Value	Significance
Model	3.41	9	0.38	35.28	<0.0001	**
*X*_1_	1.23	1	1.23	114.92	<0.0001	**
*X*_2_	0.19	1	0.19	17.35	0.0042	**
*X*_3_	0.51	1	0.51	47.56	0.0002	**
*X*_1_*X*_2_	0.026	1	0.026	2.39	0.1663	–
*X*_1_*X*_3_	0.036	1	0.036	3.37	0.1092	–
*X*_2_*X*_3_	*0.022*	1	*0.022*	2.10	0.1908	–
$X_{1}^{2}$	0.47	1	0.47	43.93	0.0003	**
$X_{2}^{2}$	0.023	1	0.023	2.18	0.1834	–
$X_{3}^{2}$	0.79	1	0.79	74.12	<0.0001	**
Residual	0.075	7	0.011			
Lack of fit	0.029	3	0.010	0.84	0.5398	–
Pure error	0.046	4	0.012			
Cor. total	3.48	16				
*C.V.*% = 1.86%						
*R*^2^ = 0.9784						
*R*^2^*_adj_* = 0.9507						

“–” means no significant; * *p*-Value <0.05 significant; ** *p*-Value <0.01 highly significant.
